# The multidimensional impact of malnutrition in incurable cancer

**DOI:** 10.1097/SPC.0000000000000817

**Published:** 2026-07-07

**Authors:** Amy McLuskie, Richard JE Skipworth, Anne Finucane

**Affiliations:** a Edinburgh Cancer Research Centre, University of Edinburgh, Edinburgh, UK; b Clinical Surgery, Royal Infirmary of Edinburgh, University of Edinburgh, Edinburgh, UK; c Clinical Psychology, Health in Social Science, University of Edinburgh, Edinburgh, UK; d Marie Curie Hospice, Edinburgh, UK

**Keywords:** cachexia, caregiver, dietary counselling, incurable cancer, malnutrition, nutrition impact symptoms

## Abstract

**Purpose of review:**

The physiological consequences of malnutrition are well-established, yet the disruption to psychological well-being, identity and interpersonal relationships is less known and equally significant. This review focuses on recent multidimensional evidence on malnutrition in incurable cancer, particularly the psychological, social and relational effects.

**Recent findings:**

Malnutrition and cachexia carry physical, psychological and relational consequences. For those with incurable cancer, this can lead to social isolation. Furthermore, it can also cause distress, guilt, anxiety and conflict around encouraging intake between those with incurable cancer and their caregivers. Qualitative studies indicate unmet needs resulting from reduced access to specialised nutritional care. Individualised dietary counselling is one of the most effective interventions for managing nutritional intake and addressing individual needs and concerns, thereby improving quality of life.

**Summary:**

Those living with incurable cancer face complex nutritional challenges that affect their physical health, emotional well-being and interpersonal relationships. Tailored nutritional care can offer benefits aside from weight gain – it can reduce anxiety, improve quality of life and support adjustment and family understanding, which could help alleviate eating-related distress in those with incurable cancer. Recent findings highlight that individualised nutritional care – encompassing the management of nutrition impact symptoms, emotional support, alignment with personal goals and caregiver support – is essential to high-quality, supportive and palliative care. Nutritional screening and care should be integrated into oncological care, addressing holistic multimodal nutritional needs.

## INTRODUCTION

Malnutrition and cachexia are highly prevalent among people living with incurable cancer, with significant physical, psychological and relational consequences [1–4^▪^]. Cachexia causes involuntary weight loss, muscle wasting, systemic inflammation and limited response to conventional nutritional support [5^▪^^▪^,^6^]. Estimates suggest that cancer-related malnutrition and cachexia affect between 40 and 80% of this population, with higher rates in lung, pancreatic, gastrointestinal and head and neck cancers [5^▪^^▪^,^6^–^11^^▪^^▪^].

Malnutrition and cachexia can have similar presentations but are distinct conditions, with these terms often being used interchangeably. Malnutrition is a broad construct that results from inadequate nutritional intake, impaired nutrient utilisation and/or disease-associated inflammation. This can lead to negative changes in body composition and clinical outcomes. Cachexia is a multifactorial syndrome characterised by ongoing skeletal muscle loss, with or without the loss of fat, systemic inflammation and progressive functional impairment [6].

**KEY POINTS**

- Investment in nutritional care is necessary to improve outcomes.
- The psychological and relational effects of eating difficulties emphasise the need for holistic, family-centred approaches.
- Promoting comfort and enjoyment around eating should be a priority.

This narrative review aligns with the European Society for Clinical Nutrition and Metabolism (ESPEN) definition proposed by Muscaritoli *et al.* [12], in which cachexia is described as disease-related malnutrition with inflammation. The Fearon definition defines cachexia as an ongoing loss of skeletal muscle mass, which cannot be fully reversed by nutritional support, causing functional decline. Both definitions of cachexia recognise it as a multifactorial syndrome associated with systemic inflammation, involuntary weight loss, negative energy and protein balance, resulting in adverse clinical outcomes. Despite the similarities in the definitions, Fearon’s definition emphasises the refractory loss of muscle and subsequent impairment [6]. The ESPEN definition places cachexia in the broader spectrum of disease-associated malnutrition [12].

Malnutrition remains an under-recognised and under-treated clinical condition despite its substantial impact on morbidity, functional decline, treatment tolerance and survival [4^▪^,^10^,^13^]. Nutrition impact symptoms, including anorexia, taste changes, nausea and fatigue, can worsen malnutrition and cause delays in treatment, increased hospital admissions and impaired quality of life [9,11^▪^^▪^,^14^^▪^]. It is estimated that 20% of cancer deaths are attributable to the effects of malnutrition [1_,_^15^].

The physiological consequences of malnutrition are well known, yet the disruption to psychological well-being, identity, and interpersonal relationships is less known and equally significant. For patients and families, loss of appetite, progressive weight loss and the inability to eat as before may be experienced as distressing markers of decline and impending mortality [8_,_^9^_,_^16^]. Conventional nutritional goals, such as weight maintenance or increased nutritional intake, might not coincide with either the realistic outcomes or the priorities of those with incurable cancer regarding nutrition. Given the complexity and multidimensional impact of malnutrition, nutritional care should form an essential part of oncology, supportive and palliative care [8_,_^17^].

This narrative review aims to examine the multidimensional impact of malnutrition in incurable cancer, including recent evidence on the physical, psychological, emotional and relational effects. It will conclude by outlining implications for clinical practice and future research.

## MULTIMODAL CARE FOR CANCER CACHEXIA

**FIGURE 1.**
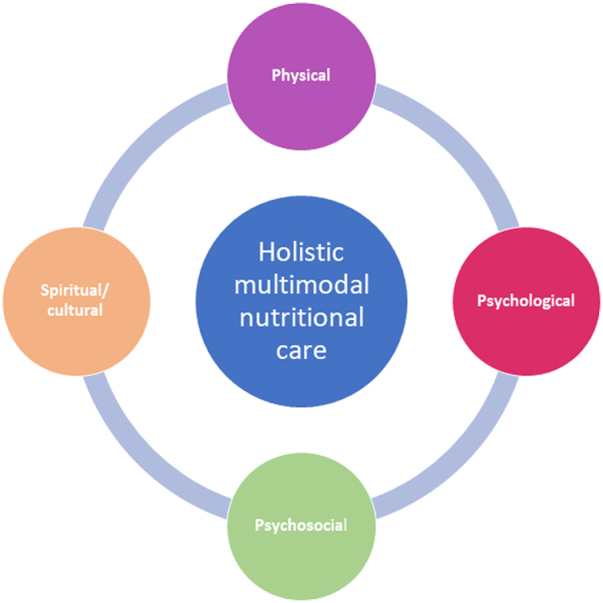
Holistic multimodal nutritional care. Adapted from Amano et al. [19].

There is accumulating evidence on the importance of holistic multimodal care for those with cancer cachexia, not just addressing the physical symptoms through nutrition, exercise and pharmacology, but also addressing the psychological needs of individuals [8], and extending beyond this into a social context, taking into account the cultural meaning of food and eating [10_,_^18^]. The holistic multimodal care model (Fig. 1) conceptualises nutritional care as a person-centred process shaped by the interaction of four domains: physical, psychological, spiritual/cultural and psychosocial. The model emphasises coordinated, multimodal care that addresses holistic, person-centred needs.

## PHYSICAL IMPLICATIONS

Malnutrition and cachexia in incurable cancer are due to a multifactorial interconnection of metabolic changes, systemic inflammation, reduced intake, disease burden and/or treatment side effects [3_,_^7^_,_^9^_,_^10^]. Weight loss and muscle wasting cause a decrease in strength, physical performance and independence, often precipitating a downward spiral of debility and treatment intolerance. Energy and protein requirements can be difficult to achieve in people with incurable cancer, which may be compounded by nutrition impact symptoms – anorexia, early satiety, dysphagia, nausea, taste changes and side effects of cancer treatments. Reduced intake due to nutritional impact symptoms diminishes the capacity to maintain nutritional status, increasing the risk of hospitalisation and poorer treatment tolerance [9_,_^20^]. Clinical outcomes, such as functional status, quality of life and survival, are consistently poorer in those with significant weight loss [3_,_^10^_,_^13^].

## PSYCHOLOGICAL CONSEQUENCES

Malnutrition carries a heavy psychological burden. Eating is usually associated with care, comfort, normality and pleasure. When people can no longer eat as they once did, this disturbance may result in emotional distress, anxiety and a feeling of loss [8_,_^9^_,_^21^]. Hopkinson *et al.*’s [21] qualitative work identified food as a ‘symbol of life’, making eating difficulties an existential challenge that can threaten identity and autonomy. Weight loss can affect body image and self-perception and may lead to social withdrawal due to embarrassment or a desire to hide physical changes [5^▪^^▪^,^22^]. Loss of control regarding eating and weight is commonly perceived as part of disease progression, reinforcing fear and anticipatory grief [8_,_^23^]. A recent qualitative study of patients, caregivers and healthcare professionals (*n* = 57) across three European countries found that eating difficulties negatively affect the quality of life for both those with cancer and their caregivers [9]. This causes distress and difficulties in coping with problems related to eating. The same study also identified that the use of commercially available, commonly sweetened, nutritional products, such as shakes, was intended to address nutritional needs of people with cancer but failed to address the more important aspect of the enjoyment of eating. This emphasises how the loss of enjoyment around eating can extend beyond the individual into the social context [9].

## SOCIAL AND RELATIONAL IMPACTS

Previous studies consistently show that food carries symbolic significance, representing love, nurture and normality [21_,_^24^]. Eating difficulties disrupt this, adding to emotional distress for both people with incurable cancer and caregivers. Those with incurable cancer may feel guilty for not eating food prepared for them, while caregivers may feel helpless or frustrated when efforts to encourage eating are unsuccessful [5^▪^^▪^,^24^]. As eating becomes more difficult, many individuals experience changes in family roles and a loss of identity. Cooking or sharing meals, which can be sources of pleasure, routine, or self-expression, may become burdensome or impossible [18]. A change in these activities not only affects the individual with incurable cancer but also alters relational dynamics within families [9]. This can also have a lasting impact on caregivers, who witness the physical changes and distress of a person struggling to eat a meal [5^▪^^▪^], highlighting the need for education from healthcare professionals.

Food is part of social practices and family life; incurable cancer disrupts roles, expectations and shared routines. Eating difficulties are experienced not only by the person with cancer but also by family members, who often take on the responsibility of preparing, encouraging and monitoring food intake [7_,_^13^_,_^24^]. They may interpret weight loss and reduced intake as signs that the person has given up or that they are failing in their caregiving role [9]. The relational impacts of eating and drinking challenges associated with incurable cancer are clearly articulated in recent studies, which describe how mealtimes become a site of tension, where pressure to eat and resistance to eating create conflict and emotional strain [13_,_^24^]. Caregivers may experience guilt, anxiety and frustration as they struggle to come to terms with the changing appetite and symptoms of the person they are caring for, often without adequate professional guidance [9_,_^15^], which can lead to further psychological distress for caregivers [7].

Cancer can lead to decreased social networks, reduced participation in social events and challenges in maintaining social ties [22_,_^25^]. Social relationships often fade as individuals with incurable cancer, and their caregivers may withdraw from situations involving food. Reasons for this can include embarrassment about appearance, fatigue and the effort required to eat or explain eating difficulties [10_,_^18^_,_^26^].

## UNMET NEEDS

Findings from previous studies have shown that nutritional needs – both physical and psychological – are frequently unmet due to limited specialised nutritional care in oncology and palliative care settings [11^▪^^▪^,^24^,^27^]. People with incurable cancer and their caregivers are often unsure about whether to continue encouraging eating, how to interpret weight loss and what to expect as the illness progresses [5^▪^^▪^,^9^]. This uncertainty contributes to anxiety, avoidance of eating and different expectations [10]. It is essential that there is clear, compassionate and consistent support and guidance from dietitians and healthcare professionals. Findings from recent studies have revealed the importance of helping those affected by changes in appetite to understand and manage their symptoms, as well as navigate relational challenges around food [9_,_^10^_,_^15^_,_^20^]. When provided, such support can ease distress, reduce conflict and nurture acceptance [28]. Nutritional advice that focuses solely on increasing calories and weight gain can feel challenging and unrealistic [9]. Nutritional care should value the meaning food holds for that person and their caregiver, taking into account changing priorities and goals [9].

## NUTRITIONAL SCREENING

There is growing recognition that nutritional screening should be embedded in routine oncology and palliative care assessments. This allows clinicians to identify malnutrition and nutrition impact symptoms that may compromise oral intake. In doing so, a proactive rather than reactive response to changes can be taken as they arise [11^▪^^▪^,^17^]. Timely nutritional screening and early identification of nutritional risk enable referral to dietetic services and are associated with improved clinical outcomes and quality of life [29–31]. Nutritional screening tools should be validated, standardised and easy to use in clinical practice [11^▪^^▪^,^32^]. Validated tools, such as the Patient-Generated Subjective Global Assessment [33] or the Malnutrition Screening Tool [3], which healthcare professionals can complete at diagnosis and continue throughout the disease trajectory, should be used as appropriate [1,11^▪^^▪^,^17^,^34^].

## NUTRITIONAL INTERVENTIONS IN THOSE WITH INCURABLE CANCER

Recent systematic reviews have shown that individualised dietary counselling can improve energy and protein intake, body composition and quality of life, including fatigue and psychological well-being [14^▪^,^35^]. Dietary counselling provided by dietitians offers opportunities to address nutrition impact symptoms, adapt eating strategies, explore food preferences and normalise expectations regarding weight and appetite. Nutritional interventions such as oral nutritional supplements, enteral nutrition and parenteral nutrition have varying levels of evidence. Oral nutritional supplements may improve nutritional intake and nutritional status and can be given alongside counselling. However, they may cause early satiety or adverse gastrointestinal symptoms, and overuse can lead to taste fatigue. This underscores the need for nutrition to be incorporated into multimodal care, rather than delivered in isolation, and for adherence to nutritional interventions to be monitored for their efficacy [35].

Nutritional care can help reduce symptom burden and improve nutritional intake, and enhance quality of life, particularly when interventions are personalised and delivered as part of multimodal care [15_,_^35^_,_^36^]. For many people, meaningful benefits can also arise even when weight gain is unlikely, as nutritional care can help alleviate anxiety, maintain enjoyment of food and offer emotional reassurance [36].

A recent longitudinal study (*n* = 160) of advanced cancer patients investigating nutritional risk across several domains of quality of life, including pain, physical and emotional functioning, found that those at nutritional risk had decreased quality of life [17]. Arakawa *et al.* [8] explored the need for multimodal care in those with cancer cachexia in palliative care settings (*n* = 170), relating to nutrition impact symptoms, oral intake, anxiety, depression and distress, to be delivered by an interdisciplinary team. Participants were divided into two groups: the high-need (*n* = 88) and low-need (*n* = 82). Those in the high-need group presented with significantly higher nutrition impact symptoms, anxiety and distress. This highlights the importance of addressing psychosocial distress caused by eating-related difficulties [8]. These studies support the need for integrated nutritional care as a vital component of supportive and palliative care, contributing to improved patient outcomes and quality of life [8_,_^17^].

Recent evidence on more invasive interventions, such as enteral or parenteral nutrition, indicates a more complex balance of benefits and harms. A scoping review of those with advanced head and neck cancer found that enteral tube feeding was initiated with the aim of reducing complications and improving nutritional and treatment outcomes, with the majority of evidence being descriptive. Some studies highlighted individuals declining enteral tube feeding due to the psychological burden. Cultural beliefs must also be addressed prior to initiating enteral tube feeding. Different religions and cultures can hold diverse values, and if these are not taken into account, they can lead to distress for those involved, emphasising the need for person-centred care when discussing enteral nutrition [10]. Artificial nutritional support offers limited benefit, except in circumstances where there is a mechanical obstruction or reversible factors impacting intake. For those in the last 3–6 months of life, evidence does not support extensive benefit, and risks include infection, discomfort and increased caregiver strain [3_,_^20^]. A retrospective analysis of patients (*n* = 68) with a malignant bowel obstruction receiving parenteral nutrition found that it could prolong survival in those with appendiceal cancer, but there was a high treatment burden and complication risk [37]. Ethical consideration is needed when initiating treatments that may later need to be withdrawn, particularly when benefits are limited. Without anticipatory conversations, there is a risk that families may interpret nutritional failure as a problem to be fixed rather than as a natural course of the disease in advanced stages [3_,_^20^]. Nutritional interventions that do not align with individual goals may undermine autonomy and contribute to family conflict.

## DISCUSSION

Despite guideline recommendations [1_,_^2^] for nutritional care, access to specialist nutritional care remains inconsistent due to limited routine screening, resource limitations and underfunding of services [11^▪^^▪^,^15^]. Dietitians integrated into oncology and palliative care pathways can help improve symptoms, reduce distress and support decision-making around complex interventions.

Research evidence converges on the importance of high-quality nutritional care in incurable cancer [9_,_^10^_,_^19^_,_^20^_,_^36^]. Identifying nutrition impact symptoms early supports timely management, helping with oral intake and quality of life [38^▪^]. When weight gain is not achievable, symptom improvement can support comfort and enjoyment of eating. Dietitians play a vital role in delivering specialist nutritional care, but all clinicians should be involved in identifying nutritional risks and providing integrated care.

Nutritional interventions must include what matters most to the person, whether this is continuing social participation, managing symptoms, or activities of daily living, such as cooking, or reducing distress around eating [20]. Goals should be individualised and realistic, particularly in those with cachexia or progressive decline, where attaining pre-illness weight is unlikely. Promoting comfort and enjoyment around eating should be a priority, with less emphasis on weight gain [20]. Management of symptoms, such as taste changes, fatigue, or nausea, with strategies can also help support oral intake. Clear, compassionate communication is necessary regarding the benefits and limitations of nutritional interventions for those with incurable cancer to prevent unrealistic expectations or a sense of failure.

This narrative review highlights the multidimensional impact of malnutrition in incurable cancer. Nutritional decline is not just a physical entity; it shapes psychological well-being, relationships and identity. While nutritional interventions may not be able to reverse advanced cachexia [39_,_^40^], they offer many benefits when tailored to individual needs and goals and grounded in clear communication. Dietary counselling, in particular, has been shown to help improve quality of life and symptom burden [20_,_^35^].

The psychological and relational effects of eating difficulties emphasise the need for holistic, family-centred approaches [38^▪^]. Patients and caregivers often experience shared distress around mealtimes, a fear of declining health and a lack of guidance regarding nutrition [9]. Integrating nutritional care into oncology and palliative services can help address unmet needs, improve quality of life and reduce family conflict [2_,_^41^_,_^42^]. Despite strong evidence and guidelines, nutritional care is inconsistently delivered. Limited access to specialist dietitians contributes to unmet needs and unnecessary emotional distress. Investment in nutritional care is necessary to improve outcomes [11^▪^^▪^].

Future research needs to explore the experiences of people with incurable cancer and their caregivers in greater depth, particularly how they make sense of nutritional changes, navigate identity loss and experience nutritional support (or lack thereof) from healthcare systems. Assessing the lived experience requires a multidimensional approach, including qualitative methods such as interviews to better understand the evolving needs of people with incurable cancer and their caregivers.

## CONCLUSION

People living with incurable cancer face complex nutritional challenges that affect physical well-being, emotional health and interpersonal relationships. Individualised dietary counselling focused on managing nutritional impact symptoms, in accordance with personal goals, and supporting caregivers should be regarded as essential to high-quality oncology, supportive and palliative care. Integrating insights from those with incurable cancer and their caregivers about what matters to them regarding nutrition and nutritional care can facilitate healthcare professionals in delivering supportive, compassionate, patient-centred care, helping to improve quality of life within the realms of a life-limiting illness. Recent evidence highlights that optimal nutritional care extends far beyond calories and weight: it requires an understanding of the meaning of food, the relational dynamics around eating and the experiences of people with cancer and caregivers throughout the course of incurable cancer.
